# High-density mapping of quantitative trait loci for grain-weight and spikelet number in rice

**DOI:** 10.1186/s12284-014-0014-5

**Published:** 2014-08-19

**Authors:** Dong-Min Kim, Hyun-Sook Lee, Soo-Jin Kwon, Mark Edward Fabreag, Ju-Won Kang, Yeo-Tae Yun, Chong-Tae Chung, Sang-Nag Ahn

**Affiliations:** Department of Agronomy, College of Agriculture & Life Sciences, Chungnam National University, Daejeon, 305-764 South Korea; National Academy of Agricultural Science, Rural Development Administration, Suweon, 441-707 South Korea; Chungnam Agricultural Research and Extension Services, Yesan, 340-861 South Korea; Present address: Department of Variety Testing, Korea Seed & Variety Service, Ministry of Agriculture, Food and Rural Affairs, Kimcheon, 740-220 South Korea

**Keywords:** Rice, Spikelets per panicle, Grain weight, QTL, Linkage, Near isogenic line

## Abstract

**Background:**

High grain yield is one of the most important traits requiring improvement in rice breeding programs. Consequently, the genetic basis of spikelets per panicle (SPP) and grain weight (TGW) have received much research focus because of their importance in rice yield.

**Results:**

In this study, IL28, which is a near isogenic line (NIL) developed by introgressing chromosomal segments of the cultivar ‘Moroberekan’ into the cultivar ‘Ilpumbyeo’, showed a significant increase in the number of spikelets per panicle (SPP) and 1,000-grain weight (TGW) compared to the recurrent parent, Ilpumbyeo. Quantitative trait locus (QTL) analysis in 243 F2 plants derived from a cross between IL28 and Ilpumbyeo indicated that both *qSPP6* and *qTGW6* are located in the interval RM3430–RM20580. Following substitution mapping with 50 F_3:4:5_ lines, *qSPP6* was mapped to a 429-kb interval between RM20521 and InDel-1, while *qTGW6* was mapped to a 37.85-kb interval between InDel-1 and SNP--3 based on the *japonica* genome sequence. This result indicates that *qSPP6* and *qTGW6* are different genes. Yield trials with substitution lines indicated that lines harboring the homozygous Moroberekan segment at both the *qSPP6* and *qTGW6* region showed significantly higher grain yield than Ilpumbyeo.

**Conclusion:**

Because the Moroberekan alleles for SPP and TGW have been shown to be beneficial in the genetic background of Ilpumbyeo, both the *qSPP6* and *qTGW6* alleles might prove valuable in improving rice yields. Closely linked SSR markers are expected to facilitate the cloning of genes that underlie these QTLs, as well as with marker-assisted selection for variation in SPP and TGW in rice breeding programs.

**Electronic supplementary material:**

The online version of this article (doi:10.1186/s12284-014-0014-5) contains supplementary material, which is available to authorized users.

## Background

Rice (*Oryza sativa* L.) is the world’s most important cereal food crop. The anticipated rapid increase in the global human population, which is expected to reach 9.1 billion by 2050, might generate serious food shortage problems. Moreover, various factors, such as water scarcity, soil salinity, disease, climate change, and reduced arable land, will exacerbate food shortages in the next 50 years (Khush [1999] and Khush [2005]; Zhang [2007]). Therefore, researchers are focusing on increasing existing crop grain yield levels. Grain weight, spikelets per panicles, and the number of panicles per plant are the most important components of grain yield. However, the genetic analysis of these three yield components is difficult, because these traits are controlled by multiple genes, in addition to being influenced by the environment. Therefore, the advent of molecular maps for rice (Causse *et al*. [1994]) and quantitative trait locus (QTL) analysis approaches have facilitated the analysis of these quantitative traits.

Genes/QTLs for spikelets per panicle (SPP) have been reported in populations derived from inter-specific crosses (Xiong *et al*. [1999]; Thomson *et al*. [2003]; Suh *et al*. [2005]; Li *et al*. [2006] and Linh *et al*. [2008]), *indica*-*indica* crosses (Lin *et al*. [1996]; Zhuang *et al*. [1997]), and inter-subspecific crosses (Yamagishi *et al*. [2002]; Ando *et al*. [2008]). To date, a few QTLs associated with SPPs have been detected, including *Gn1a*, *APO1*, *DEP1*, and *OsSPL14. Gn1a* controls grain number in rice, and was found to encode cytokinin oxidase/dehydrogenase (OsCKX2), which is an enzyme that degrades the phytohormone cytokinin (Ashikari *et al*. [2005]). Higher expression of OsSPL14 during the reproductive stage of rice promotes panicle branching leading to higher grain yield (Miura *et al*. [2010]). *DEP1*, which alters panicle architecture, and hence increases grain yield, has also been identified (Huang *et al*. [2009]). Another approach to identify the genes related to panicle architecture is through the analysis of mutants that alter panicle structure. The characterization of the aberrant panicle organization 1 (*apo1*) mutant in rice revealed that *APO1* positively controls spikelet number by suppressing the precocious conversion of inflorescence meristems to spikelet meristems (Ikeda *et al*. [2007]). Ookawa *et al*. ([2010]) also reported that a near isogenic line carrying *APO1* enhanced culm strength and increased spikelet number per panicle.

Grain weight (GW) is another characteristic that is targeted to enhance rice yield. Many QTLs for grain size have been identified in rice populations based on crosses between divergent cultivars or accessions (Lin *et al*. [1996]; Cui *et al*. [2003]; Ishimaru [2003]; Zhang *et al*. [2009]). Several genes that regulate grain weight have been identified, including *GS3*, *GS5*, *GW5*, *GW2*, and *TGW6. GW2* is a QTL for rice grain width and weight, which encodes a previously unknown RING-type E3 ubiquitin ligase that negatively regulates cell division by targeting its substrate(s) for degradation by the ubiquitin-proteasome pathway (Song *et al*. [2007]). Ishimaru *et al*. ([2013]) found that when the IAA-glucose hydrolase gene stops functioning, TGW6 enhances grain weight through pleiotropic effects on source organs, and leads to significant yield increases.

Numerous studies have reported that one genomic region harbors more than two independent genes that are closely linked, or a gene with pleiotropic effect on several traits (Xie *et al*. [2008]; Jin *et al*. [2009]; Lou *et al*. [2013]; Ji *et al.,*[2014]). Xie *et al*. ([2008]) reported that two QTLs, sn9.1 and gw9.1, for 1,000-grain weight and spikelets per panicle were identified in the 37.4-kb interval flanked by markers RM24718 and RM30005. Two QTLs acted in an additive manner in this region, resulting in the NIL with the *O. rufipogon* alleles exhibiting significantly higher SPP and TGW values compared to the recurrent parent, Hwaseongbyeo (Xie *et al*. [2008]). However, it remains unclear whether either of the two closely linked QTLs, or one pleiotropic QTL, are associated with the observed variation. A large-sized population combined with high-density mapping is required to determine whether target traits are controlled by closely linked QTLs, or one pleiotropic QTL. In previous studies (Ju *et al*. [2008]), IL28 was developed, which is an introgression line derived from a cross between the cultivars Ilpumbyeo and Moroberekan, showing higher SPP and TGW values compared to Ilpumbyeo. Kim *et al*. ([2013]) reported that a QTL for spikelets per panicle was located between RM20521 and RM20572 using F_2:3_ populations derived from a cross between Ilpumbyeo and IL28. The present study was carried out to detect a novel QTL for TGW, and to clarify its relationship to the *qSPP6* QTL using F_3:4:5_ populations.

## Results

### Characteristics of IL28

**Figure 1:**
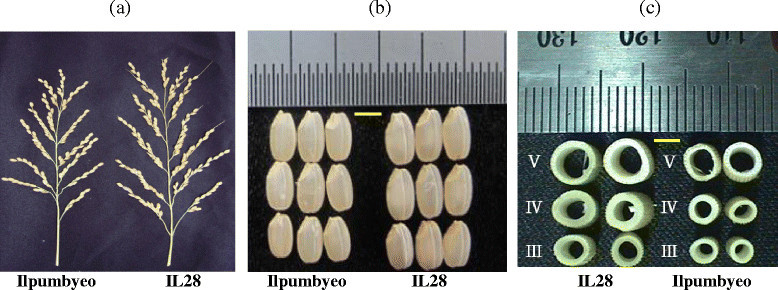
**Phenotype difference between Ilpumbyeo and IL28 in (a) panicle, (b) grain size (scale bar: 3 mm), and (c) node width (scale bar: 3 mm)**. III, IV, and V indicate the third, fourth, and fifth nodes, respectively.

**Figure 2:**
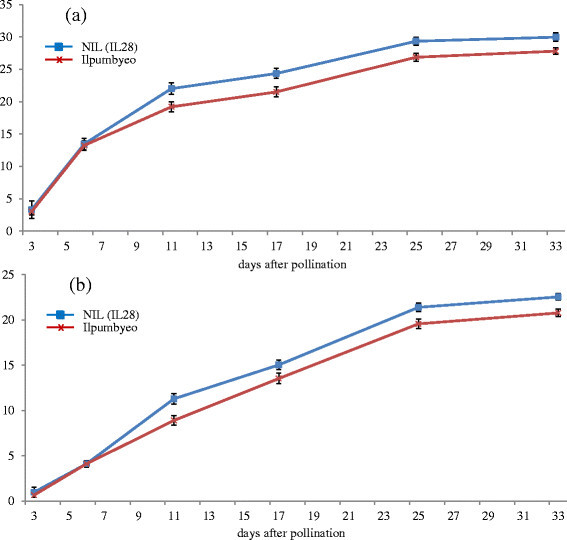
**Characterization of grain filling in Ilpumbyeo and IL28. (a)** Time-course of endosperm fresh weight increase. **(b)** Time-course of endosperm dry weight increase. Data are mean ± s.d. (n = 7 to 10 plants) in **a** and **b**.

In our previous study (Kim *et al*. [2013]), the genetic map of IL28 was constructed using 134 markers that exhibited polymorphism between Ilpumbyeo and Moroberekan, with 2 Moroberekan introgression segments being detected on chromosomes 4 and 6. The phenotypic evaluation of agronomic traits for the introgression line, IL28, and the recurrent parent was conducted at two locations, Daejeon and Yeasan, in 2013. Comparison of 10 agronomic traits between IL28 and Ilpumbyeo obtained in the current study are shown in Table 1 and Figure 1. The results indicated that there were highly significant differences (*P* < 0.01) in panicle length (PL), secondary branch number (SBN), spikelets per panicle (SPP), first node width (FNW), second node width (SNW), and 1,000-grain weight between IL28 and Ilpumbyeo, while no significant difference was obtained for tiller number (TN) and culm length (CL). Because IL28 has a large endosperm and heavier grain, we investigated the grain milk-filling rate in IL28 and Ilpumbyeo. No difference was observed in either endosperm fresh weight or dry weight at 3 d and 6 d after fertilization (Figure 2). In comparison, both the fresh and dry weight of IL28 were significantly higher (*P* < 0.01) compared to those of Ilpumbyeo at 11 d after fertilization. These differences reached a maximum ~25 d after fertilization, at which point the fresh and dry weight of the endosperm of IL28 were 11.2% and 13.4% higher compared to Ilpumbyeo, respectively. Thus, an increase in grain weight in IL28 which is associated with higher grain milk-filling rate might be due to the Moroberekan segment on chromosome 6 considering that no QTL for grain width and weight was detected in the Moreberekan segment on chromosome 4 .

**Table 1:** **Comparison of 10 agronomic traits between IL28 and Ilpumbyeo at two locations in 2013**

Traits	Mean ± S.D.					
	Daejeon		Difference A and B	Yeasan		Difference A and B
	Ilpumbyeo(A)	IL28(B)		Ilpumbyeo(A)	IL28(B)	
Days to heading	115	113	NS	113	112	NS
Grain weight/5 plants (g)	175	189	** ^#^	174	190	** ^#^
Panicle length (cm)	22.3 ± 1.3	24.3 ± 2.0	**	22.5 ± 1.6	25.4 ± 1.8	**
Spikelets per panicle	157 ± 11	182 ± 13	**	152 ± 14	185 ± 15	**
Secondary branch	25.3 ± 4.4	32.2 ± 1.9	**	28.3 ± 3.4	34.2 ± 2.2	**
First node width (mm)	4.3 ± 0.2	5.2 ± 0.2	**			**
Second node width (mm)	5.3 ± 0.2	6.2 ± 0.2	**			**
1,000 grain weight (g)	21.6 ± 0.2	22.9 ± 0.3	**	21.5 ± 0.3	23.1 ± 0.2	**
Amylose content (%)	18.1 ± 0.1	18.3 ± 0.1	NS	18.2 ± 0.1	18.2 ± 0.1	NS
Protein content (%)	5.5 ± 0.1	5.8 ± 0.1	NS	5.8 ± 0.1	5.7 ± 0.1	NS

Data were from the trial 2013 in Daejeon and Yeasan. Comparison of two lines was carried out at Daejeon for three years from 2011 to 2013, and the results were nearly the same. Only the 2013 data from Daejeon are presented.

-Not evalutated

^#^ **Significant at *P* < 0.01, NS: not significant.

### Trait variation of the F_2_ population

In our previous study (Kim *et al*. [2013]), four traits (panicle length, numbers of primary branches and secondary branches, and spikelets per panicle) exhibited continuous and normal distributions in the 243 F_2_ plants. The 250-grain weight of Ilpumbyeo and IL28 was 5.51 and 6.14, respectively. In all traits measured, transgressive plants with higher or lower mean values compared to either parent were observed. The correlation between spikelets per panicle and grain weight was not significant (*r* = 0.102).

### QTL analysis

Three significant QTLs for panicle length, secondary branch number, and spikelets per panicle were detected. These QTLs occurred in the same region, near the SSR marker RM3430 (Kim *et al*. [2013]). In addition, the QTL for grain weight was detected in the same region, on chromosome 6. The QTL for grain weight explained 8.3% of phenotypic variance. The Moroberekan alleles increased all trait values of panicle length, secondary branch number, spikelets per panicle and grain weight at this region.

### Substitution mapping of *qTGW6*

**Figure 3:**
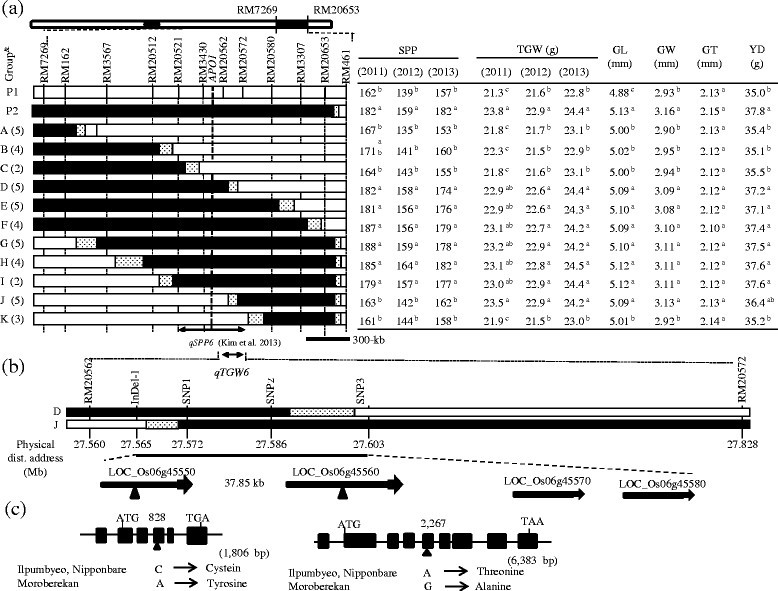
**Development of a high density map of** ***qTGW6*** **and structures of candidate genes for** ***qTGW6.*** **(a)** Graphical representation of F_3:4:5_ lines and a fine scale map of the target region on chromosome 6. *Apo1* located between RM3430 and RM20562 might be allelic to *qSPP6* detected in this study. White and black portions of the graph are homozygous Ilpumbyeo, homozygous Moroberekan, respectively and dotted regions are where crossing-over occurred. Genotypes of the 44 lines were double checked at F_3_ generation. The table to the right of the graphical genotypes indicates mean values of traits. Numbers followed by the same letter in each column are not significantly different at *P* = 0.05 based on Duncan’s multiple range test. & No. in ( ) indicate the number of F_3_ lines. P_1_: Ilpumbyeo, P_2_: IL28. **(b)** High density map of *qTGW6* using two groups (D and J) and four markers. Four genes were predicted in the target 37.85-kb region. The arrowheads indicate the position of a single non-synonymous nucleotide substitution. **(c)** Structures of two genes, Loc_Os06g45550 and Loc_Os06g45560. Loc_Os06g45550 consists of five exons (black boxes) and four introns and Loc_Os06g45560 consists of 8 exons and 7 introns. Numbers on the structure of two genes show the position of non-synonymous nucleotide substitutions from the start codon.

To confirm and narrow down the target region containing *qTGW6*, 1,120 F_2_ plants derived from a cross between Ilpumbyeo and IL28 were genotyped with 4 additional markers near the target region (RM20512, RM20521, RM20562, and RM20572) and one marker (RM551) located on chromosome 4. A total of 44 F_2_ homozygous plants with different cross-over breakpoints between RM7269 and RM20653 on chromosome 6 were selected and selfed to produce F_3:4:5_ lines. Genotype analysis indicated that these 44 F_2_ plants did not have Moroberekan segment on chromosome 4. According to the marker genotype, the 44 F_3:4:5_ lines were divided into 11 groups, and used for the substitution mapping of *qTGW6* (Figure 3). The mean phenotypic values of each trait for each F_3:4:5_ line was compared to those of Ilpumbyeo and IL28 at the *P* < 0.05 level. In a previous study, the QTLs for SPP, PL, SBM, FNW, and SNW were consistently mapped between RM20521 and RM20572, which are 680-kb apart (Kim *et al*. [2013]). For the *qTGW6* locus, groups D, E, F, G, H, I, and J showed significantly higher values than Ilpumbyeo for 1,000-grain weight, whereas no significance difference was detected between Ilpumbyeo and groups A, B, C, and K in 2011, 2012, and 2013 (Figure 3a). Thus, *qTGW6* was located downstream of RM20521 and upstream of RM20580. Group D showed significantly higher values for SPP affected by *qSPP6* and for TGW compared to Ilpumbyeo, while group J was significantly different from Ilpumbyeo for TGW but not SPP. This result indicates that *qSPP6* and *qTGW6* are different genes. Comparison of the genotypes of recombinants delimited the *qTGW6* between markers RM20562 and RM20572, based on the finding that the TGW of both D and J groups were significantly different from that of Ilpumbyeo. The region between the two markers RM20562 and RM29572 for groups D and J shared common segment harboring of *qTGW6*. To further confirm the common segment shared by groups D and J, we genotyped the F_3_ lines using four markers (one InDel marker and three SNP markers) located between RM20562 and RM20572. The genotypes showed that *qTGW6* was defined in a 37.85-kb region between InDel-1 and SNP3 (Figure 3b).

### Evaluation of the grain shape trait

To determine which grain shape traits are associated with increase in grain weight, three grain shape traits (specifically, grain length [GL], width [GW], and thickness [GT]) were evaluated for the two parents and F_5_ lines (Figure 3). The mean phenotypic values of each F_5_ line were compared to those of Ilpumbyeo and IL28 at the *P* < 0.05 level. The GL of Ilpumbyeo and IL28 were 4.88 mm and 5.13 mm, respectively. The GW values for Ilpumbyeo and IL28 were 2.93 mm and 3.15 mm, respectively. The GL and GW values of IL28 were significantly higher compared to Ilpumbyeo (*P* < 0.05), whereas no significant difference was detected for GT. The GL and GW of the groups with *qTGW6* (D, E, F, G, H, I, and J) were significantly different from those of the groups without *qTGW6* (A, B, C, and K). This finding indicates that *qTGW6* variation is caused by variation in GL and GW at *qTGW6*. Grain weight was significantly correlated with GL and GW (*P* < 0.01 for both characteristics), but not with GT (*r* = −0.06).

### Cross-section of the central part of the spikelet hull

**Figure 4:**
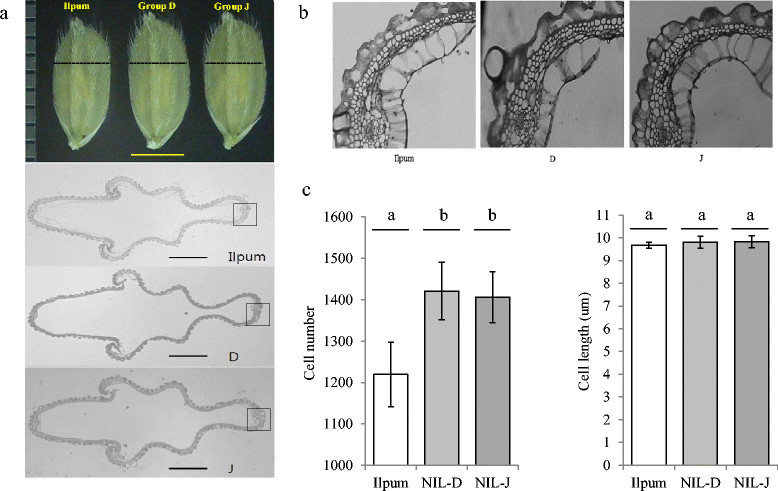
**Histological analyses of spikelet hull 3 days before heading in Ilpumbyeo, group D, and group J. (a)** Cross-section of spikelet hull. Upper: spikelets (scale bar, 3 mm). Low: cross-section of spikelet hull (scale bar, 500um). Dotted line indicates position of cross-section **(b)** Magnified view of spikelet hull cross-section boxed in a. **(c)** Comparison of total cell number and mean cell length in the outer glume cell layers of spikelet hull of Ilpumbyeo, group D, and group J. (n = 5 to 8 spikelets). Numbers followed by the same letter on each box are not significantly different at *P* = 0.05 based on Duncan’s multiple range test.

Given that the hull of the IL28 spikelet was wider than that of Ilpumbyeo before fertilization, the cross-section of the central part of the spikelet hull for groups D and J was analyzed in comparison to Ilpumbyeo to investigate the origins of the observed size differences (Figure 4). The outer glume cell layer of groups D and J contained substantially more cells (16.5% and 15.3%, respectively) compared to that of Ilpumbyeo, with only a 1.3% and 1.4% increase in cell length, respectively. These data indicate that the increased width of group D and J spikelet hulls mainly results from an increase in cell number, rather than cell size, indicating that *qTGW6* may be involved in the regulation of cell division.

### Impact of the QTL cluster on yield per plant

Eleven groups containing 44 F_5_ lines were used for the yield trials, together with Ilpumbyeo and IL28 in 2013 (Figure 3). IL28 had significantly higher grain yield compared to Ilpumbyeo. IL28 produced 8% higher grain yield on average compared to Ilpumbyeo. All groups with the Moroberekan segment at both *qSPP6* and *qTGW6* (i.e., groups D, E, F, G, H, and I) had significantly higher grain yield values compared with the groups without the Moroberekan segment at both *qSPP6* and *qTGW6* (i.e., groups A, B, C, and K) and Ilpumbyeo (Figure 3). Also, the yield per plant of group J with *qTGW6* and without *qSPP6* was higher than that of group C and Ilpumbyeo at *P* < 0.1 (*P* = 0.068, *P* = 0.067, respectively), and this result implies the contribution of *qTGW6* to the yield increase. However, we could not determine the effect of *qSPP6* on yield due to the lack of line(s) only with the Moroberekan segment at *qSPP6*.

### Candidate genes for *qTGW6*

*qTGW6* was defined in a 37.85-kb region between InDel-1 and SNP3. On the basis of available sequence annotation databases (http://www.gramene.org), there are four predicted genes in the target region. The functional annotations of the four genes are as follows: Loc_Os06g45550 (five exons and four introns) and Loc_Os06g45560 (eight exons and seven introns) are retrotransposon protein putative expressed genes; Loc_Os06g45570 (one exon) is a VQ domain-containing protein putative expressed gene; Loc_Os06g45580 (one exon) is a RING-H2 zinc finger putative protein expressed gene (Figure 3). We compared the genomic sequences of the open reading frame (ORF) and promoter region of the four genes between Ilpumbyeo and Moroberekan. Comparison of the genomic sequence of two genes (Loc_Os06g45550 and Loc_Os06g45560) among Nipponbare, Ilpumbyeo, and Moroberekan revealed the existence of a single non-synonymous SNP in the third and fourth exon of Loc_Os06g45550 and Loc_Os06g45560, respectively. The non-synonymous SNP at the third exon of Loc_Os06g45550 is characterized by the nucleotide Cytosine in Ilpumbyeo and the nucleotide Adenine in Moroberekan. The change in the nucleotide base from Cytosine to Adenine resulted in missense mutation from cysteine to tyrosine. The non-synonymous SNP at the fourth exon of Loc_Os06g45560, is characterized by the nucleotide Adenine in Ilpumbyeo and the nucleotide Guanine in Moroberekan. The nucleotide substitution A (adenine) to G (guanine) in Loc_Os06g45560 resulted in a missense mutation from Threonine to Alanine. No sequence variation in open reading frames and the promoter region of two genes was detected for Loc_Os06g45570 and Loc_Os06g45580 among the three varieties, Nipponbare, Ilpumbyeo, and Moroberekan.

## Discussion

There has been much debate as to whether similar genomic locations of QTLs that affect different traits are caused by the pleiotropy of a single gene or the tight linkage of several genes that individually influence specific traits. The question of pleiotropy versus tight linkage might be resolved by using a large-size population combined with high-density mapping, because two linked QTLs segregate independently in NIL-F_2:3_ populations. Recently, several QTLs controlling the same or multiple traits have been confirmed to be closely linked in a small genomic region. For example, the rice photoperiod sensitivity gene, *Hd3* was originally detected as a heading date-related quantitative trait locus that is localized on chromosome 6 in the F_2_ population (Yano *et al*. [1997]). For the fine-scale mapping of *Hd3*, high-resolution linkage analysis using a large segregating population derived from advanced backcross progeny between the *japonica* rice variety, Nipponbare and the *indica* variety, Kasalath, was carried out. As a result of the high-resolution linkage mapping of *Hd3*, two tightly linked loci, *Hd3a* and *Hd3b*, which promote flowering under short-day conditions and inhibit heading under long-day conditions, were identified in the *Hd3* region (Monna *et al*. [2002]). Lou *et al*. ([2013]) mapped two QTLs (*qSPP5* and *qTGW5*) for spikelets per panicle and 1,000-grain weight to the same region on chromosome 5. Substitution mapping with the BC_5_F_3_ and BC_5_F_4_ populations derived from a cross between QTL-NIL and the recurrent parent, demonstrated that two tightly linked QTLs (*qSPP5* and *qTGW5*) were different. Several QTLs with large pleiotropic effects on multiple traits have also been found and cloned (*APO1*, *Ghd7*, and *Ghd8* or *DTH8*). For instance, Hua *et al*. ([2002]) reported that a QTL cluster influences multiple traits (namely, grain per panicle, plant height, and heading date) on chromosome 7 in a primary population. High-resolution mapping with a large-sized segregating population and complementation tests revealed that these three traits are controlled by a single locus, designated as *Ghd7* (Xue *et al*. [2008]).

The original target of this study was *qSPP6*, which was mapped on the long arm of chromosome 6 (Ju *et al*. [2008]). In the process of the fine-scale mapping, the QTL for additional yield components (including panicle length, node width, and 1,000-grain weight) were consistently detected in the same region. A total of 243 F_2_ plants derived from a cross between NIL (IL28) and Ilpumbyeo for genetic analysis was not large enough to clarify the relationship between SPP and TGW. To confirm the precise location of the two QTLs, substitution mapping was carried out. Following substitution mapping with 44 F_3:4:5_ lines, *qSPP6* was mapped to a 431-kb interval between RM20521 and SNP1, while *qTGW6* was mapped to a 37.85-kb interval between InDel-1 and SNP3, based on the *japonica* genome sequence. Therefore, the development of NIL and substitution mapping has enabled us to map SPP and TGW at the fine-scale.

No TGW-associated QTL was detected near the *qTGW6* QTL in this study, indicating that *qTGW6* is a novel QTL for TGW. Among the four predicted genes in the target region between InDel-1 and SNP-3 (37.85-kb), Loc_Os06g45570 encodes a VQ domain-containing protein. VQ domains are believed to be critical for the defense response to pathogens by interacting with WRKY transcription factors (Tao *et al*. [2011]). Loc_Os06g45580 encodes the RING-H2 zinc finger protein. Recent studies reported a critical role of the ubiquitin pathway in grain development in rice (Song *et al*. [2007]; Weng *et al*. [2008]). *GW2*, a major QTL for rice grain width and weight encodes a new RING-type E3 ubiquitin ligase. *GW5*, another major QTL underlying rice width and weight, is likely to act in the ubiquitine-proteasome pathway to regulate cell division during cell division (Weng *et al*. [2008]). *qTGW6* exhibited common function for grain width with *GW2* and *GW5* in that it was associated with increased cell numbers, a wider spikelet hull and an increased grain milk-filling rate. This raises a possibility that Loc_Os06g45580 is a candidate for *qTGW6*. However, no sequence variation in open reading frames and the promoter regions of two genes, Loc_Os06g45570 and Loc_Os06g45580 was observed between Ilpumbyeo and Moroberekan and this result seems to suggest that they are not candidate genes for *qTGW6.* The other two genes in the target region are retrotransposon proteins, Loc_Os06g45550 and Loc_Os06g45560. Loc_Os06g45560 was transcribed in seedling and embryo/endosperm 25 days after pollination but Loc_Os06g45550 was not transcribed based on the available gene expression database (RGAP, http://rice.plantbiology.msu.edu/expression.shtml). More than 40% of rice genome is composed of repetitive sequences and transposable elements (Goff et al. [2002]). A recent study showed that the expression of Tos17, a copia-like retrotransposon was affected by overexpression or down-regulation of several rice SUVH (Su(var)3–9 homologs) genes and RNAi plants with down-regulated SDG728, a rice SUVH gene showed a reduced seed size phenotype (Qin *et al*. [2010]). This raises a possibility that a relationship between altered seed morphology and overexpression of Tos17 might exist. Interestingly, our alignment analysis of *GW5* a.a sequence using BLASTP program showed that *GW5* protein has high homology with two retrotransposon proteins encoded by LOC_Os08g26160.1 and LOC_Os05g47720.1 (data not shown), although it has been reported that the protein encoded by the *GW5* ORF had no sequence homology with any proteins of known molecular function (Weng *et al.*, [2008]). *GW5* protein showed 56% homology to the retrotransposon protein with 194 a.a encoded by *LOC_Os08g26160.1* and 47-50% homologies to the transposon protein with 411 a.a. by *LOC_Os05g47720.1*. Numerous Indels and single-nucleotide polymorphisms (SNPs) were detected between Nipponbare (*japonica)* and 9311 (*indica)* and Indels are mainly caused by transposons (Feng *et al*. [2002]). Indels and SNPs in some key domestication genes including *qSH1* and *GW2* are responsible for morphological changes occurred during domestication (Konishi *et al*. [2006]; Song *et al*. [2007]). Also, the difference in grain width between *japonica* and *indica* cultivars is associated with a 1212-bp deletion harboring the *GW5* ORF which was possibly caused by transposons (Weng *et al*. [2008]). These observations support our hypothesis that the protein encoded by the *GW5* ORF might be a retrotransposon and Loc_Os06g45560 is a candidate for *qTGW6*. However, the presence of SNPs in the upstream regions of the genes between two parents suggests the possibility that a long-distance regulation is involved in controlling the *qTGW6* (data not shown). Konishi *et al*. ([2006]) reported that the casual mutation in qSH1 underlying the seed shattering was found in the 12-kb upstream region from the ORF of the gene and caused the absence of the abscission layer in Nipponbare. Additional fine mapping experiments are underway to determine the functional relationship between *qTGW6* and *GW5* in controlling seed development. Also, loss-of-function mutant lines of Loc_Os06g45560 will be evaluated for grain morphology traits and the gene expression. These informations on the molecular mechanism of *qTGW6* would be necessary to get more insights into the mechanism of grain development.

Although the candidate gene of *qTGW6* and its genetic mechanism was not elucidated here, we found that the Moroberekan chromosomal segment at this region (a tropical *japonica*) harbors QTLs for yield component traits leading to an yield increase in *japonica* cultivar. Yield trials confirmed that introgression lines containing the Moroberekan segment at the *qSPP6* and *qTGW6* loci produced significantly higher yields than Ilpumbyeo (*P* < 0.05). The grain yield per plant in IL28 was 8% higher than that of Ilpumbyeo. Moroberekan alleles in the target region on chromosome 6 had a favorable effect on yield, by increasing the number of spikelets per panicle and 1,000-grain weight, in addition to lodging tolerance by increasing the node width, whereas it had no negative effect on heading date and plant height. Thus, the *qSPP6* and *qTGW6* alleles are potentially valuable for improving rice yield. It is proposed that NILs containing the target segment associated with positive QTLs from Moreberekan should be developed from this population, and evaluated in a wide range of environments, to assess the interaction between QTLs and the environment. Tightly linked SSR markers are expected to facilitate the cloning of genes underlying these QTLs, in addition to marker-assisted selection for variation in the SPP and TGW in rice breeding programs.

## Conclusion

In this study, we demonstrated that two QTLs, *qSPP6* for spikelets per panicle (SPP) and *qTGW6* for 1,000-grain weight (TGW) are tightly linked on chromosome 6. Because Moroberekan alleles for the SPP and TGW have been shown to be beneficial in the genetic background of Ilpumbyeo, the *qSPP6* and *qTGW6* alleles may prove valuable for improving the yield potential of japonica rice cultivars. Tightly linked SSR markers are expected to facilitate the cloning of genes underlying these QTLs, in addition to marker-assisted selection for variation in SPP and TGW in rice breeding programs.

## Methods

### Plant materials

In a previous study (Kim *et al*. [2013]), IL28 was shown to exhibit a higher number of spikelets per panicle and heavier grain compared to the recurrent parent, and contained the Moroberekan chromosomal segment at the *qSPP6* region on chromosome 6; hence, it was selected for the fine-scale mapping of *qSPP6* in the current study. IL28 was crossed with the cultivar Ilpumbyeo, with the resulting F_1_ plants being self-pollinated to obtain an F_2_ population (234 plants in 2009 and 1,150 plants in 2010). To confirm *qSPP6*, 44 F_2_ homozygous plants with different cross-over breakpoints between RM7269 and RM20653 were selected and selfed to produce F_3_ lines for the phenotyping and substitution mapping of *qSPP6*. In the process of the fine-scale mapping of *qSPP6*, a QTL for 1,000-grain weight (TGW) was consistently detected in the same region. To confirm the precise location of *qSPP6* and *qTGW6*, these 44 F_3_ lines were advanced to F_4_ and F_5_ lines, which were used to evaluate spikelets per panicle, grain weight, grain morphology traits, and grain yield.

### Field trial and trait evaluation

A total of 243 F_2_ plants and 1150 F_2_ plants (2009 and 2010), and 41 F_3:4:5_ lines (2011, 2012, and 2013) and the parental lines were grown at the experimental field of Chungnam National University, Daejeon, Korea. In 2009, the 243 plants and the parents were planted at distances of 30 × 15 cm, and evaluated for grain (brown rice) weight (150 out of 243 plants were used to evaluate grain weight). In 2010, for the substitution mapping of *qTGW6*, 1,150 F_2_ plants were genotyped with four additional markers being located between RM7269 and RM20653 (RM20512, RM20521, RM20652, and RM20572) and one marker (RM551) located on chromosome 4. The experiment using 44 F_3:4:5_ lines derived from 44 F_2_ plants followed a completely randomized block design with two replications, one row per plot, and 25 plants per row in 2011, 2012, and 2013. The middle 10 plants from each line were chosen for the evaluation, and the average of the measurements was used for the phenotype of each line for the spikelets per panicle (SPP) and 1,000-grain weight (TGW). SPP were measured by averaging the three major panicles per plants. Grains with hulls were allowed to dry naturally after harvesting, and partial or unfilled seeds were removed by soaking grains in water. Fully filled seeds were re-dried in an oven at 30°C for 24 h. The TGW was evaluated by measuring the weight of 250 randomly selected, de-hulled grains per plant (10 plants per line). In 2013, grain length (GL), grain width (GW), grain thickness (GT), and yield per plants (YD) were measured. The grain length (GL), grain width (GW), and grain thickness (GT) were measured for 50 grains (brown rice) per plant (10 plants per line) using a 150-mm Vernier caliper (Mitutoyo Corp., Japan). Yield per plant (YD) was measured by averaging the grain yield (g) of 10 plants that were randomly selected from the center of each plot per block. The 1,000-grain weight and the yield per plant were corrected for the 12% grain moisture content.

### DNA extraction and SSR analysis

DNA was extracted from the leaf tissue of the F_2_ population using a chloroform-based DNA extraction protocol (Causse et al., [1994]). A 20-μL reaction mixture was used, containing 5.0 μL (5 ng/μL) of template DNA, 0.1 μl (5 Unit/μL) Taq polymerase, 0.8 μl dNTP (2.5 mM each), Forward + Reverse primer 1 μl (10 pmol each), 2.0 μl 10× PCR buffer (10 mM Tris–HCl PH 8.3, 50 mM KCl, 1.5 mM MgCl2, 0.1% gelatin), and 11.1 μl triple-distilled water. Amplification was achieved using a Thermo Cycler (Bio-Rad) based on the step-cycle program set for denaturation at 94°C for 5 min, subsequent denaturation was performed at 94°C for 1 min, annealing at 55°C for 1 min, extension at 72°C for 1 min; steps 2 to 4 were repeated for a total of 35 cycles, with a final extension step at 72°C for 5 min. PCR products were run on 4% polyacrylamide denaturing gels for 1–2 h at 1800–2000 V, and marker bands were revealed by silver staining (Panaud *et al*. [1996]). The orientation of the SSR markers was based on the SSR maps (McCouch *et al*. [2002]). Due to the lack of markers showing polymorphism in the region between RM20562 and RM20572, additional genotyping of the F_3_ lines was conducted with four targeted SNPs and one Insertion/Delition marker. To detect the SNPs and InDels of Ilpumbyeo and Moroberekan, resequencing with NGS (Next-Generation Sequencing) was conducted using the two parents according to Jeong *et al*. ([2013]). Through the comparison of genomic sequences corresponding to the region between RM20562 and RM20572, a total of 28 polymorphisms (14 SNPs, and 14 Insertion/Deletions) were detected between Ilpumbyeo and Moroberekan. The polymorphisms were assayed by the direct sequencing of the four regions. Information on the primers used is presented in Table 2. The primers were designed according to the Nipponbare sequence (http://rgp.dna.affrc.go.jp/E/IRGSP/Build5/build5.html). The first InDel, hereafter referred to as InDel-1, occurs at the 27,565,774th position based on the Nipponbare sequence (www.gramene.org), and is characterized by nucleotide - (1 bp deletion) in Ilpumbyeo and nucleotide A in Moroberekan. The first SNP, SNP-1, that occurs at the 27,572,767th position, is characterized by nucleotide G in Ilpumbyeo and nucleotide A in Moroberekan. The second SNP, SNP-2, that occurs at the 27,586,202th position, is characterized by nucleotide A in Ilpumbyeo and nucleotide G in Moroberekan. The third SNP, SNP-3, that occurs at the 27,629,080th position, is characterized by nucleotide A in Ilpumbyeo and nucleotide C in Moroberekan.

**Table 2:** **Primer sequences of newly developed SNP markers**

Marker name	Forward primer (5’-3’)	Reverse primer (5’-3’)	Position	Product size (bp)
InDel-1	GAGAAAGTGACCCTCGTTTAGT	CAAGACATGACACTACGATTGC	27,565,623 – 27,565,899	276
SNP1	CCATATGATTAAATGGGAGGCTC	ACATAGGCACTGCCAAGGTTC	27,572,630 – 27,572,896	266
SNP2	ACGGACGCCTGCTATAGGGA	CACCAGAGGCGATCTTCTTC	27,585,994 – 27,586,360	366
SNP3	GAATATCACGATCAAAGAACTTG	GTTACTCTAATATGATAATATGCC	27,603,470 – 27,603,822	352

### Statistical analysis

Statistical analysis was performed using Qgene software version 2.30 for Macintosh (Nelson [1997]) and SAS (SAS Institute). Single point analysis (SPA) was performed to determine the effect of each marker on each trait. In SPA, a QTL was confirmed if the phenotype was associated with a marker locus at *P* < 0.005, or with two adjacent marker loci at *P* < 0.01. The proportion of total phenotypic variation explained by each QTL was calculated as an R^2^ value, from the regression of each marker/phenotype combination. QTLs were mapped at a fine-scale by comparing the phenotypic means of two genotypic classes of recombinants (Ilpumbyeo homozygote and Moroberekan homozygote) within the target region by using the SAS statistical software package.

### Histological analysis

Paraffin-embedded sections of spikelet samples were prepared according to Li *et al*. ([2009]), with minor modifications. Plant materials were fixed in FAA (50% ethanol, 5% glacial acetic acid and 5% formaldehyde) and stored at 4°C for 24 h. The fixed spikelets were dehydrated by soaking them for 6 h in a gradient ethanol series (60%, 70%, 80%, 90%), and were then incubated in 100% ethanol overnight. Dehydrated spikelets were embedded in Paraplast (Sigma). Tissue sections (8 μm thick) were cut using a rotary microtome, transferred onto gelatin-coated glass slides, and dried at 42°C for 1 day. The sections were de-paraffinized with 100% xylene for 5 min, followed by soaking for 2 min in 50% ethanol/50% xylen, 100% ethanol, and sterile water. The samples were stained with toluidine blue for 30 s, and washed twice with sterile water. The samples were then soaked for 2 min in 70% ethanol, 80% ethanol, 90% ethanol, and 100% ethanol. Finally, the samples were cleaned by soaking them twice in 100% xylen. The sections were photographed under an Olympus BH2-RFCA using a U-PMTVC camera.

## Authors’ original submitted files for images

Below are the links to the authors’ original submitted files for images. Authors’ original file for figure 1 Authors’ original file for figure 2 Authors’ original file for figure 3 Authors’ original file for figure 4
