# Supplementary figures and images for: High-density mapping of quantitative trait loci for grain-weight and spikelet number in rice
Source: Rice (N Y). 2014 Aug 19;7:14. doi: 10.1186/s12284-014-0014-5 (PMC4884038; doi:10.1186/s12284-014-0014-5)

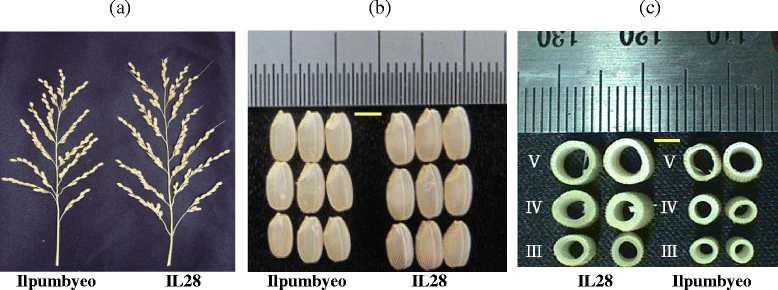

Supplement: Supplementary file 1 — Authors’ original file for figure 1 [file 12284_2014_14_MOESM1_ESM.gif]

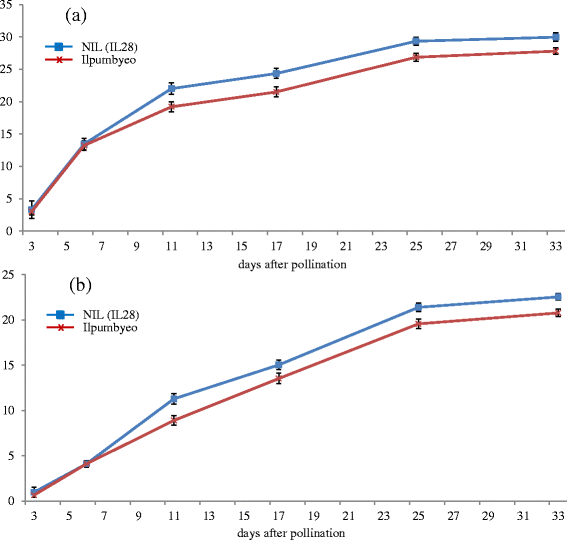

Supplement: Supplementary file 2 — Authors’ original file for figure 2 [file 12284_2014_14_MOESM2_ESM.gif]

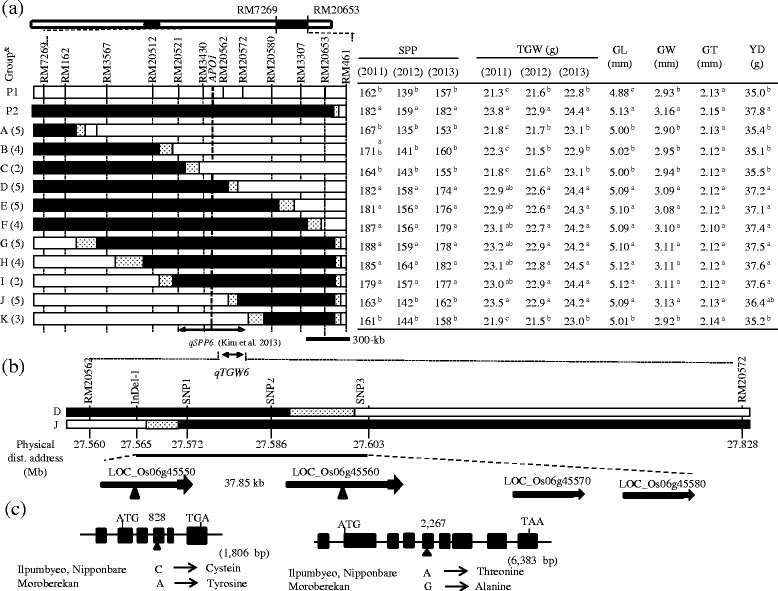

Supplement: Supplementary file 3 — Authors’ original file for figure 3 [file 12284_2014_14_MOESM3_ESM.gif]

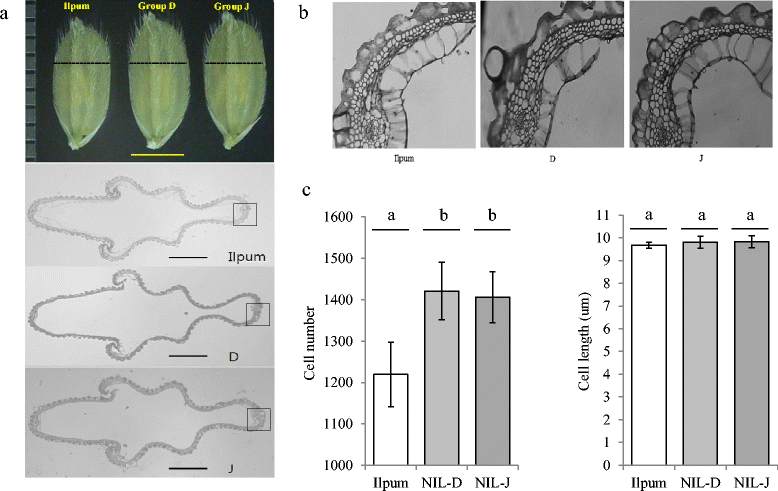

Supplement: Supplementary file 4 — Authors’ original file for figure 4 [file 12284_2014_14_MOESM4_ESM.gif]
